# Identification of Mitochondrial-Related Prognostic Biomarkers Associated With Primary Bile Acid Biosynthesis and Tumor Microenvironment of Hepatocellular Carcinoma

**DOI:** 10.3389/fonc.2021.587479

**Published:** 2021-04-01

**Authors:** Tao Zhang, Yingli Nie, Jian Gu, Kailin Cai, Xiangdong Chen, Huili Li, Jiliang Wang

**Affiliations:** ^1^ Department of Anesthesiology, Union Hospital, Tongji Medical College, Huazhong University of Science and Technology, Wuhan, China; ^2^ Department of Dermatology, Wuhan Children’s Hospital (Wuhan Maternal and Child Healthcare Hospital), Tongji Medical College, Huazhong University of Science and Technology, Wuhan, China; ^3^ Department of Gastrointestinal Surgery, Union Hospital, Tongji Medical College, Huazhong University of Science and Technology, Wuhan, China

**Keywords:** hepatocellular carcinoma, mitochondria, prognosis, bile acid, tumor microenvironment

## Abstract

Hepatocellular carcinoma (HCC) is one of the leading causes of tumor-associated deaths worldwide. Despite great progress in early diagnosis and multidisciplinary tumor management, the long-term prognosis of HCC remains poor. Currently, metabolic reprogramming during tumor development is widely observed to support rapid growth and proliferation of cancer cells, and several metabolic targets that could be used as cancer biomarkers have been identified. The liver and mitochondria are the two centers of human metabolism at the whole organism and cellular levels, respectively. Thus, identification of prognostic biomarkers based on mitochondrial-related genes (Mito-RGs)—the coding-genes of proteins located in the mitochondria—that reflect metabolic changes associated with HCC could lead to better interventions for HCC patients. In the present study, we used HCC data from The Cancer Genome Atlas (TCGA) database to construct a classifier containing 10 Mito-RGs (ACOT7, ADPRHL2, ATAD3A, BSG, FAM72A, PDK3, PDSS1, RAD51C, TOMM34, and TRMU) for predicting the prognosis of HCC by using 10-fold Least Absolute Shrinkage and Selection Operation (LASSO) cross-validation Cox regression. Based on the risk score calculated by the classifier, the samples were divided into high- and low-risk groups. Gene set enrichment analysis (GSEA), gene set variation analysis (GSVA), t-distributed stochastic neighbor embedding (t-SNE), and consensus clusterPlus algorithms were used to identify metabolic pathways that were significantly different between the high- and low-risk groups. We further investigated the relationship between metabolic status and infiltration of immune cells into HCC tumor samples by using the Cell-type Identification By Estimating Relative Subsets Of RNA Transcripts (CIBERSORT) algorithm combined with the Tumor Immune Estimation Resource (TIMER) database. Our results showed that the classifier based on Mito-RGs could act as an independent biomarker for predicting survival of HCC patients. Repression of primary bile acid biosynthesis plays a vital role in the development and poor prognosis of HCC, which provides a potential approach to treatment. Our study revealed cross-talk between bile acid and infiltration of tumors by immune cells, which may provide novel insight into immunotherapy of HCC. Furthermore, our research may provide a novel method for HCC metabolic therapy based on modulation of mitochondrial function.

## Introduction

Hepatocellular carcinoma (HCC) is one of the most common malignant cancers and is currently the fifth and seventh leading cause of cancer-related deaths worldwide in females and males, respectively (1). Despite the great progress that has been made in early diagnosis and multidisciplinary tumor management, the long-term prognosis remains poor. Therefore, novel and effective prognostic models are needed to improve clinical management by identifying patients at high risk of a poor prognosis. Conventional models use clinical TNM (Tumor, Node, Metastases) stage, vascular invasion, and other parameters to help predict the prognosis of HCC patients. However, considering the high morphological and biological heterogeneity of HCC, the efficacy of these predictive models remains unsatisfactory.

Mitochondria are centers of cellular metabolism that regulate metabolite and energy flow essential for cell growth, proliferation, differentiation, and death (2). Therefore, mitochondria are deeply involved in various cancer-related biological processes, including cancer initiation, growth, invasion and metastasis, recurrence, and resistance to drugs (3). Mutation and epigenetic modulation of mitochondrial DNA, reprogramming of energy metabolism, and changes in mitochondrial channels have been found to play vital roles in cancer biology (3). Recent studies have demonstrated that mitochondrial metabolism is a potential target for cancer therapy (4) since various mitochondrial metabolic processes are altered in tumors (5). Thus, it vitally important to take mitochondrial-related biomarkers into account when developing novel predictive tools.

The liver is the key regulator of whole-body metabolism and maintains metabolic homeostasis. Recent studies have demonstrated that substantial metabolic changes are associated with various types of cancers, including HCC (6). Thus there are potential advantages of mitochondrial-related genes (Mito-RGs) as prognostic biomarkers for HCC. Therefore, exploration of underlying metabolic changes in HCC may bring new insights that could improve the prognosis of HCC patients.

In the present study, we constructed a classifier containing 10 Mito-RGs for HCC cell survival by utilizing Least Absolute Shrinkage and Selection Operation (LASSO) Cox regression. Based on the risk score calculated by the classifier, the samples were divided into low- and high-risk groups. We further investigated changes in metabolism and metabolic subgroups of HCC samples by Gene Set Variation Analysis (GSVA), t-distributed Stochastic Neighbor Embedding (t-SNE), and consensus clusterPlus. Additionally, we used the Cell-type Identification By Estimating Relative Subsets Of RNA Transcripts (CIBERSORT) algorithm and the Tumor Immune Estimation Resource (TIMER) database to investigate the relationship between metabolic status and infiltration of HCC samples by immune cells. Our results demonstrate that the Mito-RGs-based classifier can be used as a reliable predictor of HCC patient survival. The suppression of metabolic processes governing bile acid biosynthesis may play a vital role in the development and poor prognosis of HCC, providing a potential approach to treatment. Moreover, our research reveals cross-talk between bile acid and infiltration of tumors by immune cells, which may provide novel insight into immunotherapy of HCC. Therefore, our research may provide a novel method for HCC metabolic therapy based on modulation of mitochondrial function.

## Materials and Methods

### Data Source and Pre-Processing

**Figure 1:**
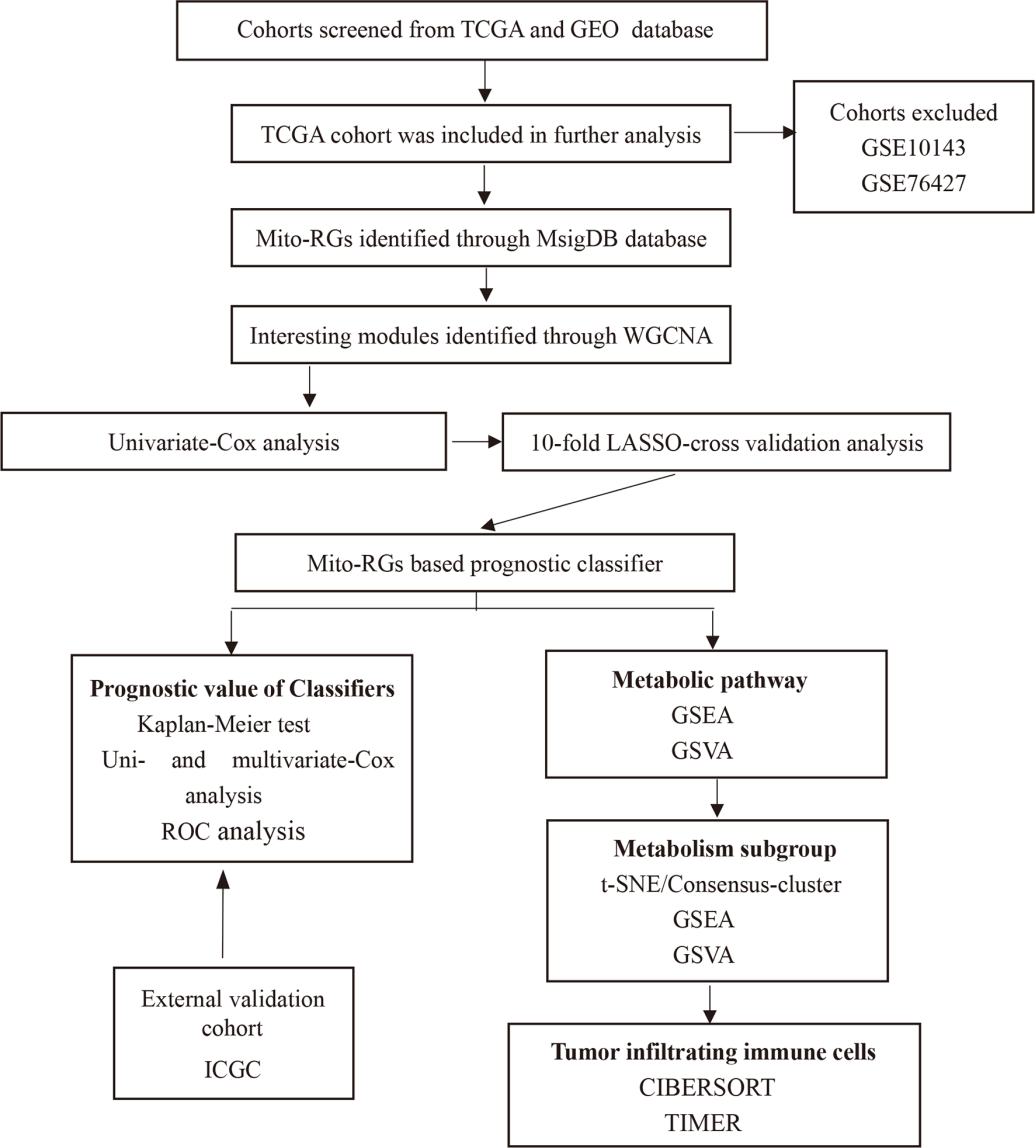
Flowchart of the construction of the Mito-RGs-based prognostic classifier. Mito-RGs, mitochondrial-related genes; ROC, receiver operating characteristic; WGCNA, weighted gene co-expression network analysis; TCGA, The Cancer Genome Atlas; LASSO, The least absolute shrinkage and selection operation; GSVA, Gene set variation analysis; GSEA, Gene set enrichment analysis.

Bioinformatics analyses were performed using the procedure shown in **Figure 1**. HCC cohorts with survival data were obtained from several databases, including GEO (Gene Expression Omnibus), TCGA (The Cancer Genome Atlas), and ICGC (International Cancer Genome Consortium). The Cancer Genome Atlas-Liver Hepatocellular Carcinoma (TCGA-LIHC) and ICGC-Liver Cancer-RIKEN-Japan (LIRI-JP) cohorts were also used for the analysis. Cohort GSE76427 from the GEO database was excluded because there was significant censoring in the survival data: 14.8% of patients were censored within 1 month, 35.7% within 1 year, and 47.8% within 2 years. GSE10143 was also excluded because of a lack of expression data for many Mito-RGs.

An RNA-seq dataset and the corresponding clinical parameters of HCC tissues (n = 374) and normal liver tissues (n = 50) were downloaded from UCSC-Xena (https://xenabrowser.net/datapages/) based on information in the TCGA database. For validation, RNA-seq data and clinical information of an additional 232 HCC tumor samples were obtained from the ICGC portal (https://dcc.icgc.org/projects/LIRI-JP). HCC patients with complete survival data and RNA-seq data were included in the subsequent analysis. HCC data were annotated by the Homo_sapiens.GRCh38.84.chr.gtf (ftp.ensembl.org) file in this study.

Mito-RGs in the present study were defined as the coding-genes of mitochondrial-located proteins, including all proteins located in the mitochondrial membrane, matrix, cristae, and mitochondrial associated endoplasmic reticulum membranes. Depending on subcellular localization, all the genes were divided into 1001 Gene Ontology (GO) cellular component gene sets in the molecular signatures database (MSigDB) database (http://software.broadinstitute.org/gsea/msigdb). A total of 23 cellular component gene sets related to mitochondria and 1571 unique genes were ultimately screened as Mito-RGs (**Supplementary Table 1**).

### Weighted Gene Co-Expression Network Construction and Detection of a Module of Interest

Weighted gene co-expression network analysis (WGCNA) for all Mito-RGs in the HCC dataset was performed according to the protocols of WGCNA (7, 8), as described previously (9, 10). Briefly, we initially performed a hierarchical clustering analysis on the expression profile to exclude outliers. Subsequently, a gene co-expression similarity measure (absolute value of Pearson’s product moment correlation, *S_ij_* = *|cor(i, j)|*) was used to relate every pairwise gene-gene relationship. An adjacency matrix was then constructed using a “soft” power adjacency function *a_ij_* = Power(*s_ij_*, *β*) = |*s_ij_|^β^* where *s_ij_* is the co-expression similarity, and *a_ij_* represents the resulting adjacency that measures connection strengths. The adjacency matrix was then used to define a network distance measure, or more precisely, a measure of node dissimilarity based on a topological overlap matrix. Specifically, the topological overlap matrix is given by


$$\omega_{ij}=\frac{l_{ij}+a_{ij}}{min\{k_{i},k_{j}\}+1-a_{ij}}$$

where, $l_{ij}=\Sigma_{u\neq i}a_{iu}a_{uj}$ denotes the number of nodes to which both *i* and *j* are connected, and *u* indexes the nodes of the network. The topological overlap matrix (TOM) is given by Ω = [ω_ij_], where ω_ij_ is a number between 0 and 1 and is symmetric (i.e., ω_ij_ = ω_ji_). The rationale for considering this similarity measure is that nodes that are part of highly integrated modules are expected to have high topological overlap with their neighbors. Clusters of genes with high topological overlap were identified as “gene modules”, utilizing a measure of dissimilarity (=1−TOM).

Correlations between modules and clinical characteristics were calculated by Pearson’s correlation tests to identify modules with significant clinical meanings. The modules that exhibited high correlations with HCC clinical characteristics were selected as modules of interest for further study.

### Identification of Prognostic Mito-RGs

A univariate Cox regression was performed for all Mito-RGs in modules of interest and the genes with P < 0.05 were identified as prognostic Mito-RGs.

### Establishment of Prognostic Classifiers

Since only the TCGA cohort was enrolled in the present study, the 10-fold LASSO cross-validation Cox regression analysis was applied to all prognostic Mito-RGs for selection of the most useful prognostic biomarkers and to construct a survival-predicting classifier. LASSO is a popular method of regression with multiple dimensional parameters (11). LASSO is a penalized regression approach that estimates regression coefficients by maximizing the log-likelihood function (or the sum of squared residuals) with the constraint that the sum of the absolute values of the regression coefficients is less than or equal to a positive constant. One interesting property of LASSO is that LASSO automatically deletes unnecessary covariates, only retain the most important variables in the final model. In 10-fold cross-validation, the samples are divided into 10 subsets (folds), each time, nine subsets are used to train the model, and then the remaining subset is used as the validation set. Finally, the 10 results are combined to determine the final coefficients. The prognosis risk scores were calculated based on a formula as follows:


$$\text{RiskSocre}=\Sigma ({\text{Genes}}_{\text{Cox coefficient}}\times {\text{Genes}}_{\text{expression levels}})$$

We then used the cutoff of the median risk score to divide the HCC patients into low- and high-risk groups. The predictive ability of the model for the training and validation cohorts, which was randomly split in a 1:1 ratio, as well as for the total cohort, was evaluated using the Kaplan-Meier log-rank test. Furthermore, the application value of the model was tested by Receiver Operating Characteristic (ROC) curve analysis, and by univariate and multivariate Cox regression analysis.

### Pathway Enrichment Analysis

In order to investigate any changes in mitochondrial function and metabolic pathways between high- and low-risk groups, we performed Gene Set Enrichment Analysis (GSEA) and Gene Set Variation Analysis (GSVA).

GSEA is a method for determining whether a given gene set is significantly enriched in a list of gene markers ranked by their correlation with a phenotype of interest. The first step of GSEA is to sort genes according to the degree of differential expression in the two sample phenotypes (normal and tumor tissues in this study). Then, the GSEA method calculates an Enrichment Score (ES) by proceeding through the list, increasing a cumulative sum when a gene is in the gene set and decreasing it if a gene is not. According to the ES, we can estimate the degree of enrichment of a gene set for the phenotype. Furthermore, GSEA normalizes the ES for each gene set to account for the variation in gene set sizes, yielding a normalized enrichment score (NES) (12). The clusterProfiler R package (13) was used to perform GSEA analysis based on the Kyoto Encyclopedia of Genes and Genomes (KEGG) database between high- and low-risk groups. KEGG analysis used a cutoff value of P < 0.05.

GSVA is a gene set enrichment method that estimates variation of pathway activity over a sample population in an unsupervised manner (14). GSVA transforms a gene expression matrix into a gene set enrichment matrix, facilitating the identification of differentially activated gene sets for each sample. We selected C2.CP.KEGG.V7.1.symbols.gmt file consisting of 186 KEGG gene sets as the reference gene set file. Then, the GSVA package was used to obtain GSVA scores for each gene set of each sample, which yielded their degree of absolute enrichment. After that, we used the limma and pheatmap packages to display distinct pathways between the high- and low-risk groups.

### Metabolic Subgrouping

T-SNE is one of the most effective methods to reduce dimensionality while maintaining the similarity between low-dimensional descriptors and high-dimensional data. In the t-SNE method, the low-dimensional space maintains the pair-wise similarity to the high-dimensional space, leading to a clustering in the embedding space close to the clustering in the high-dimensional space without losing significant structural information (15, 16). Consensus clustering is a method for unsupervised clustering that provides evidence of quantitative and visual stability for estimating the number of unsupervised classes in a dataset (17).

To deduce the metabolic status of a sample, we used the t-SNE and Consensus ClusterPlus R packages (parameters: reps = 1000, pItem = 0.8, pFeature = 1) to cluster HCC samples into different metabolic subgroups.

### Estimating Immune Cell Infiltration

In order to further explore the relationship between metabolic status and immune cell infiltration, the CIBERSORT algorithm (18) was used to estimate the fraction of 22 immune cell types in the HCC samples from gene expression data. In addition, the correlation between gene expression and tumor-infiltrating immune cells was analyzed using the TIMER database (19), a comprehensive resource for systematic analysis of immune infiltrates across multiple cancer types.

### Statistical Analysis

All statistical analyses were conducted by R version 3.6.1 (http://www.R-project.org) and GraphPad Prism 8.0 statistical software (GraphPad Software, Inc., La Jolla, CA, USA). The correlation between risk score and clinicopathological characteristics was analyzed by the chi-square test. The statistical significance of normally distributed variables of the two sample groups was estimated by the two-tailed unpaired *t*-test. P<0.05 were considered statistically significant.

## Results

### Co-Expression Network Construction and Prognostic Module Detection

**Figure 2:**
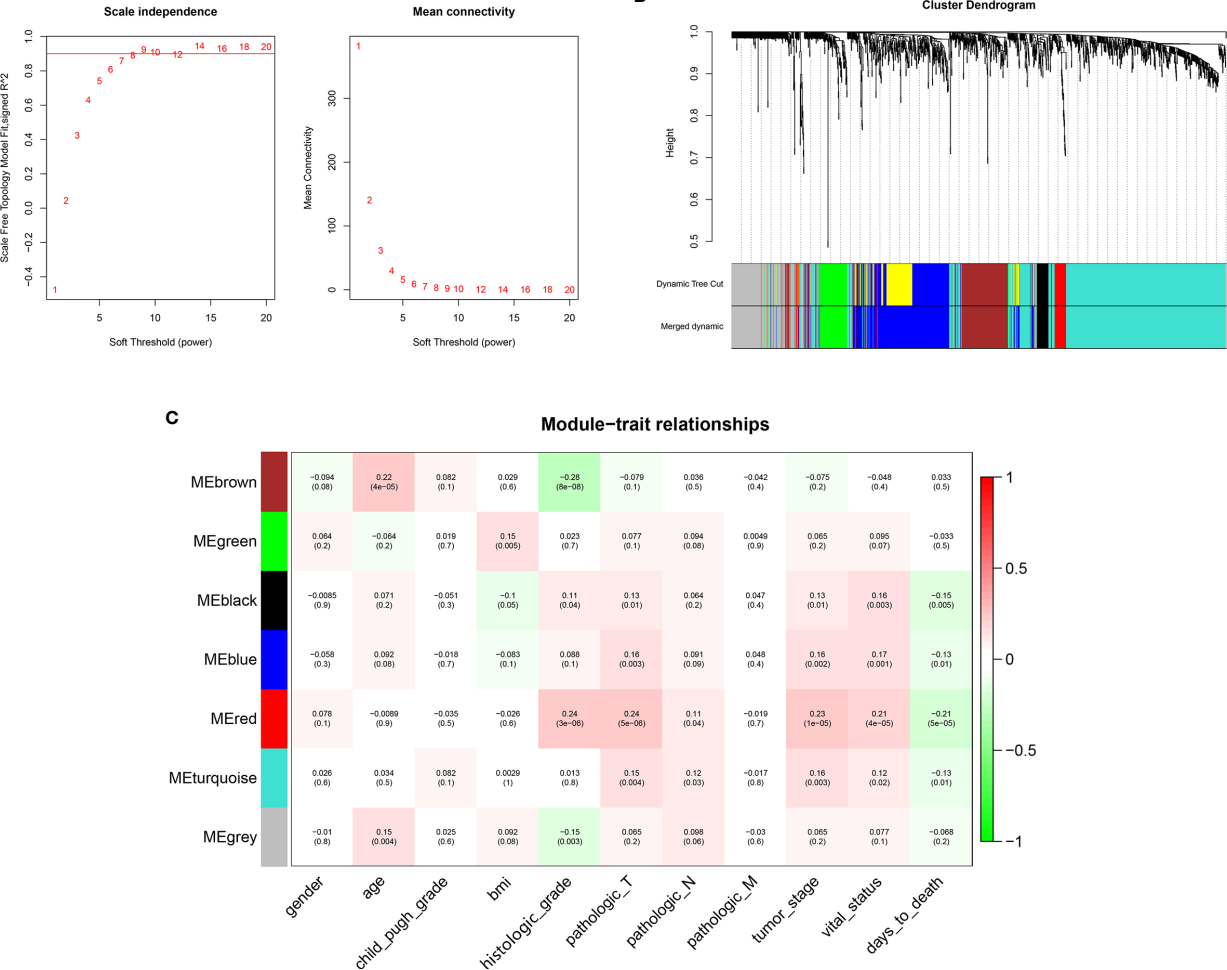
WGCNA network and module detection. **(A)** Selection of the soft-thresholding powers. Power 8 was chosen because the fitted index curve flattened out upon reaching a high value (>0.9). **(B)** Cluster dendrogram and module assignment for modules from WGCNA. The colored horizontal bar represents the modules. **(C)** Correlation matrix for Eigengene values and clinical features. Each cell includes the corresponding correlations and the *p*-values.

WGCNA was conducted on 1519 Mito-RGs in the 374 HCC samples. When the soft-threshold power β was set to 8, a scale-free network distribution was formed of the connectivity between genes in the gene network (**Figure 2A**). Then, seven co-expressed modules were identified (**Figure 2B**). The correlations between modules and clinical features, such as gender, age, Child-Pugh grade, BMI, histologic grade, pathologic T, pathologic N, pathologic M, tumor stage, vital status, and days to death were calculated. The red module was highly correlated with histologic grade (r = 0.24, P = 3 × 10^−6^), pathologic T (r = 0.24, P = 5 × 10^−6^), tumor stage (r = 0.23, P = 1 × 10^−5^), vital status (r = 0.21, P = 4 × 10^−5^), and days to death (r = −0.21, P = 5 × 10^−5^) (**Figure 2C**). Thus, the red module was selected as a prognostic module of interest to be studied in subsequent analyses.

### Identification of Prognostic Mito-RGs in a Prognostic Module

Univariate Cox regression was conducted for all Mito-RGs in the red module (n = 63) (**Supplementary Table 2**). The results showed that 50 of the 63 genes were significantly associated with overall survival (OS) of HCC patients (P < 0.001) and were therefore identified as prognostic Mito-RGs. All 50 genes (ABCC8, ACOT7, ADPRHL2, ANKZF1, ATAD3A, ATAD3B, BAK1, BMF, BSG, CAPN10, CCNB1, CDK1, COA1, COX19, DLGAP5, DTYMK, E2F1, FAM72A, FANCG, FEN1, FLVCR1, FUNDC1, FUNDC2, GARS, HJURP, HKDC1, KCNJ11, LIG1, MRM2, MRPL18, MRPL53, MTFR2, NDUFA4L2, NUDT1, OGG1, PDK3, PDSS1, PIF1, PRELID2, RAD51, RAD51C, SLC25A45, TOMM34, TOMM5, TRMU, TYMS, VAT1, XRCC3, YKT6, AC006538.1) were found to be associated with unfavorable prognosis of HCC with hazard ratios (HRs) > 1 (**Supplementary Figure 1**).

### Construction of a Prognostic Mito-RGs–Based Classifier

**Figure 3:**
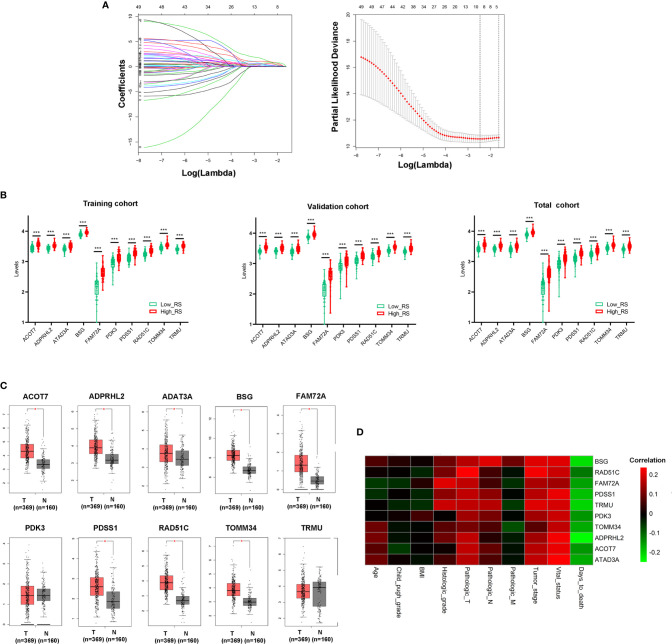
Construction of Mito-RGs-based prognostic classifier. **(A)** Results of the LASSO Cox regression suggested that all 10 genes were essential for the classifier. **(B)** Expression levels of all 10 genes of the classifier in the high- and low-risk groups from the training, validation, and total cohorts. **(C)** Expression of the 10 genes in the classifier between HCC (T) and normal liver tissues (N) in GEPIA (http://gepia.cancer-pku.cn/) based on the TCGA and GTEx databases. *P < 0.05. **(D)** Correlation between the 10 genes in the classifier and the clinical features of HCC. RS, risk score.

10-fold LASSO cross-validation Cox regression analysis was conducted to choose the most useful prognostic biomarkers for constructing a prognostic classifier based on the training cohort (**Figure 3A**). A total of 10 Mito-RGs (ACOT7, ADPRHL2, ATAD3A, BSG, FAM72A, PDK3, PDSS1, RAD51C, TOMM34, and TRMU) were identified as the most useful prognostic biomarkers, based on the minimum criteria to construct risk characteristics, and used the coefficients derived from the LASSO algorithm to determine risk scores for each sample (**Table 1**).

**Table 1:** The mitochondrial-related genes in the prognostic classifier associated with HCC in the TCGA data set.

Symbol	Univariate Cox regression analysis			LASSO Coefficient
	HR	95% CI	P-value	
ACOT7	34.213	6.863–170.547	0.000	0.562
ADPRHL2	293.836	41.093–2101.042	0.000	0.767
ATAD3A	51.854	9.832–273.46	0.000	0.211
BSG	60.209	8.929–405.992	0.000	1.226
FAM72A	2.484	1.521–4.056	0.000	0.369
PDK3	4.825	2.099–11.092	0.000	0.202
PDSS1	22.868	6.255–83.603	0.000	0.419
RAD51C	16.936	3.992–71.847	0.000	0.128
TOMM34	61.837	8.836–432.757	0.000	0.028
TRMU	38.376	7.287–202.108	0.000	1.033

The risk scores were calculated using the formula: risk scores =  (0.562 * expression level of ACOT7) + (0.767 * expression level of ADPRHL2) + (0.211 * expression level of ATAD3A) + (1.226 * expression level of BSG) + (0.369 * expression level of FAM72A) + (0.202 * expression level of PDK3) + (0.419 * expression level of PDSS1) + (0.128 * expression level of RAD51C) + (0.028 * expression level of TOMM34) + (1.033 * expression level of TRMU). Patients in every cohort were further divided into high- and low-risk groups at the cutoff value of the median risk score. Expression levels of every biomarker in different groups were analyzed. The results showed that the levels of all 10 biomarkers were much higher in the high-risk group than in the low-risk group (**Figure 3B**)

Additionally, the levels of ACOT7, ADPRHL2, ATAD3A, BSG, FAM72A, PDSS1, RAD51C, and TOMM34 in the classifier were much higher in HCC than in normal liver tissues (**Figure 3C**). Besides, the correlation between these genes and clinical characteristics showed that these genes were positively correlated with the tumor stage of HCC and negatively correlated with HCC prognosis (**Figure 3D**).

### Correlation Between the Classifier and Clinicopathologic Characteristics

As shown in **Table 2**, the clinical characteristics of gender, age, Child-Pugh grade, and pathologic M (pM) showed no significant differences between the high- and low-risk groups in the training, validation, or total cohorts (P>0.05). However, BMI in the validation (χ^2^ = 6.798, P = 0.009) and total (χ^2^ = 5.822, P = 0.016) cohorts, histologic grade in the training (χ^2^ = 14.321, P = 0.000), validation (χ^2^ = 10.951, P = 0.001), total (χ^2^ = 13.755, P = 0.000) cohorts, pathologic T (pT) in the training (χ^2^ = 6.376, P = 0.012) and total (χ^2^ = 4.779, P = 0.029) cohorts, pathologic N (pN) in the total (χ^2^ = 4.047, P = 0.044) cohort, and tumor stage in the training (χ^2^ = 5.176, P = 0.023) and total (χ^2^ = 6.925, P = 0.012) cohorts showed significant differences between the two groups.

**Table 2:** Correlations between risk score of the mitochondrial-related genes-based classifier with clinicopathological characteristics in the training cohort, validation cohort, and total cohort.

Parameters	Training cohort				Validation cohort				Total cohort			
	High risk	Low risk	χ^2^	P	High risk	Low risk	χ^2^	*P*	High risk	Low risk	χ^2^	*P*
Age (y)			0.908	0.341			0.088	0.766			0.958	0.439
<60	49	42			38	40			89	82		
>60	45	51			55	53			98	106		
Gender			0.014	0.905			1.328	0.249			0.990	0.320
Male	68	68			55	62			122	131		
Female	26	25			39	31			65	56		
Child-Pugh grade			0.514	0.473			0.729	0.393				
A	33	56			62	68			92	127	0.010	0.921
B and C	4	4			5	9			9	13		
BMI			0.625	0.429			6.798	**0.009**			5.822	**0.016**
≥28	60	54			79	63			151	130		
≥28	22	26			15	30			37	57		
Histologic grade			14.321	**0.000**			10.957	**0.001**			13.755	**0.000**
1–2	61	81			34	57			99	134		
3–4	32	10			58	36			85	51		
pT			6.376	**0.012**			0.097	0.756			4.779	**0.029**
1–2	60	73			72	73			131	147		
3–4	34	17			22	20			56	37		
pN			0.003	0.956			2.026	0.155			4.047	**0.044**
0	52	48			76	78			125	129		
1	1	1			2	0			4	0		
pM			–	–			0.944	0.331			0.953	0.329
0	57	56			77	78			133	135		
1	0	0			1	3			1	3		
Tumor stage			5.176	**0.023**			0.773	0.392			6.925	**0.012**
1–2	53	66			68	73			119	141		
3–4	29	16			25	20			55	35		

Bold values are statistically significant.

### Prognostic Value of the Classifier for Assessing Overall Patient Survival

**Figure 4:**
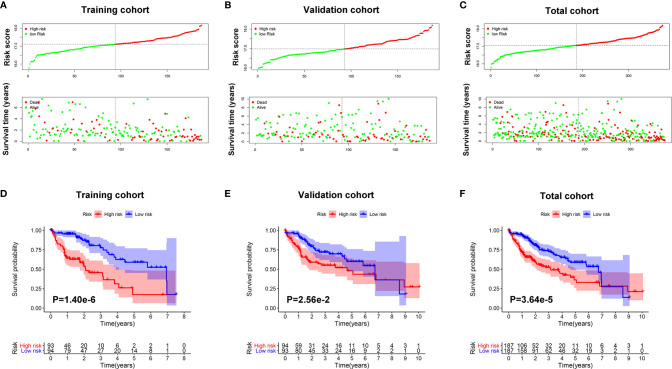
The prognostic value of the Mito-RGs-based classifier. The distribution of patients’ risk scores, survival states of the patients in the high- and low-risk groups from the training **(A)**, validation **(B)**, and total **(C)** cohorts. Kaplan–Meier survival analysis of overall survival between the high- and low-risk patients from the training **(D)**, validation **(E)**, and total **(F)** cohorts.

As shown in **Figures 4A–C**, survival time of patients decreased as risk score increased, and the number of HCC deaths also increased in the high-risk group.

To further assess the prognostic value of the classifier, a Kaplan-Meier test was conducted. As shown in **Figures 4D–F**, patients in the high-risk group had a very poor prognosis.

**Figure 5:**
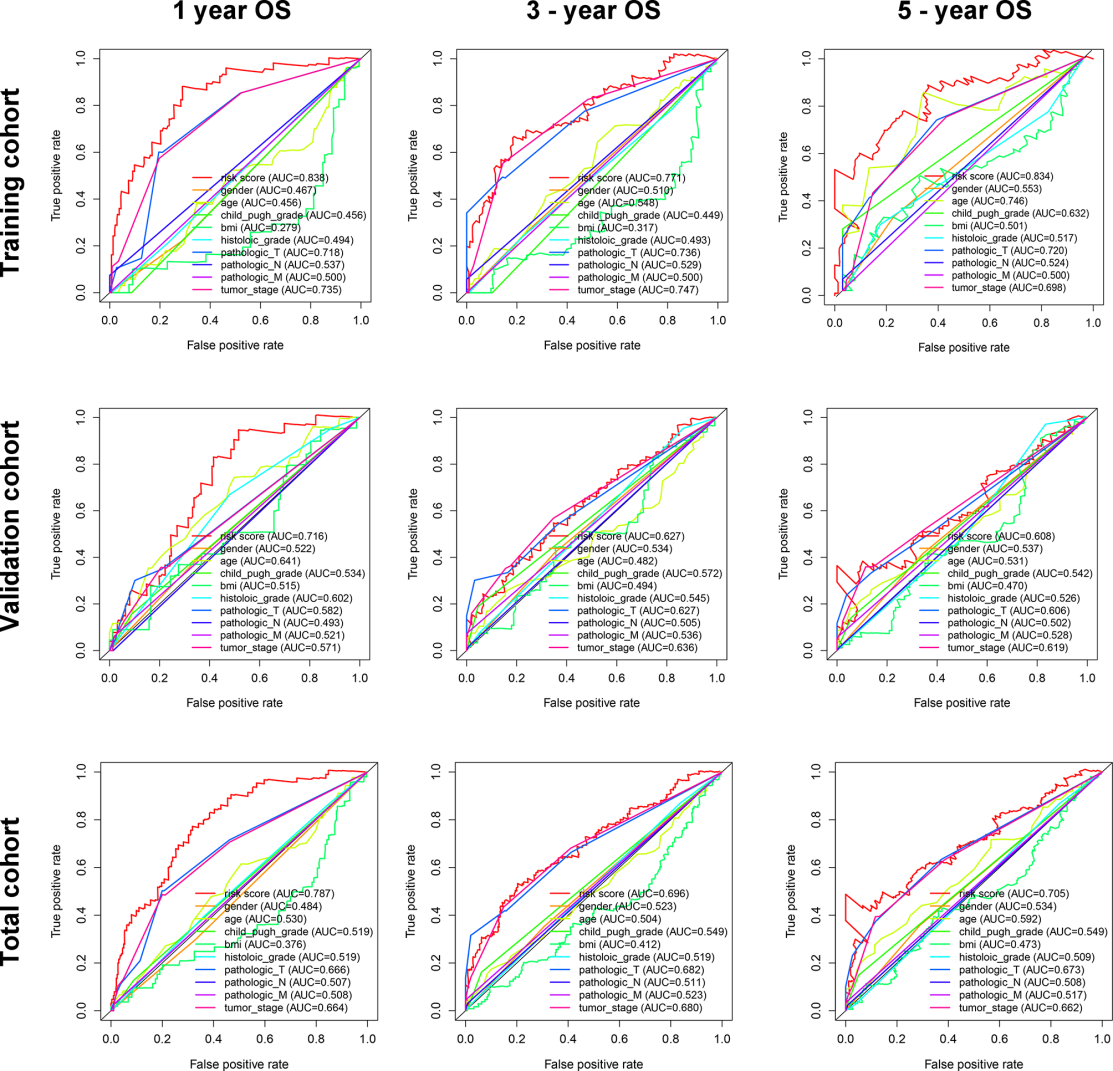
The time-dependent ROC for 1-, 3-, and 5-year overall survival predictions for the classifier in comparison with clinical features in the training, validation, and total cohorts of HCC.

In addition, in the time-dependent ROC curve analysis, the Area Under The Curve (AUC) for overall survival rates for 1, 3, and 5 years were, respectively, 0.838, 0.771, and 0.834 in the training cohort, 0.716, 0.627, and 0.608 in the validation cohort, and 0.787, 0.696, and 0.705 in the total cohort (**Figure 5**). Moreover, the predictive capability of the classifier seemed superior to histologic grade and tumor grade, which previous studies have identified as two major risk factors for tumor prognosis.

Furthermore, the results of univariate Cox regression analysis in the training, validation, and total cohorts further validated the prognostic value of the classifier (**Table 3**). Moreover, multivariate analysis suggested that the classifier included the independent risk factors for survival for HCC patients (**Table 3**).

**Table 3:** Univariate and multivariate Cox regression analyses of clinicopathologic factors for overall survival in HCC from the TCGA database.

Items	Training cohort				Validation cohort				Total cohort			
	Univariate Cox		Multivariate Cox		Univariate Cox		Multivariate Cox		Univariate Cox		Multivariate Cox	
	HR	P	HR	P	HR	P	HR	P	HR	P	HR	P
Gender	1.313	0.315			1.319	0.269			1.260	0.201		
Age	1.015	0.125			1.012	0.245			1.012	0.091		
Child grade	1.335	0.619			1.768	0.141			1.543	0.157		
BMI	0.963	0.145			1.027	0.123			1.000	0.998		
Histologic grade	1.041	0.834			1.276	0.158			1.104	0.410		
pT	2.091	**0.000**	2.809	0.127	1.358	**0.020**	1.227	0.662	1.683	**0.000**	1.655	0.213
pN	4.473	0.144			1.274	0.811			2.029	0.324		
pM	–	–			4.190	**0.017**	2.777	0.151	4.077	**0.017**	2.131	0.254
Tumor stage	2.158	**0.000**	0.612	0.495	1.363	**0.015**	1.019	0.968	1.638	**0.000**	0.955	0.913
Risk score	6.681	**0.000**	4.544	**0.000**	2.390	**0.003**	2.106	**0.031**	3.941	**0.000**	3.218	**0.000**

Bold values are statistically significant.

**Figure 6:**
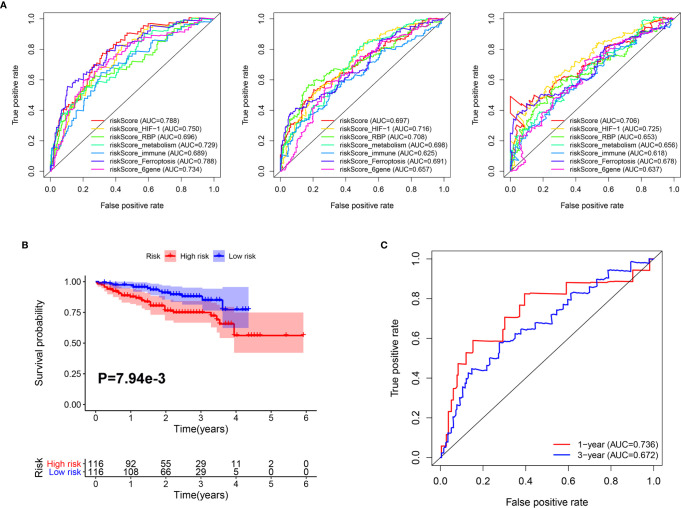
Comparison and validation of the prognostic value of the Mito-RG-based classifier. **(A)** The time-dependent ROC for 1-, 3-, and 5-year overall survival predictions for the classifier in comparison with other classifiers. **(B)** Kaplan-Meier analysis of overall survival between high and low-risk patients from ICGC cohorts. **(C)** The time-dependent ROC for 1- and 3-year overall survival predictions for the classifier in ICGC cohorts.

In addition, we compared the predictive capability of our classifier with other previously published classifiers. As shown in **Figure 6A**, the AUC of our classifier for overall survival in year 1 was higher than classifiers based on genes related to HIF-1 signaling (20), RNA binding protein (RBP) (21), metabolism (22), immune response (23), ferroptosis (24), and a six-gene–based classifier (25). For 3-year overall survival, the AUC of our classifier was higher than classifiers related to metabolism, immune response, ferroptosis, and a six-gene–based classifier. Furthermore, for 5-year overall survival, the AUC of our classifier was higher than classifiers related to RBP, metabolism, immune response, ferroptosis, and a 6-gene-based classifier.

Furthermore, to test the robustness of the classifier, the HCC patients from the ICGC cohort were also categorized into high- or low-risk groups using the median value calculated from the formula described above. As shown in **Figure 6B**, patients in the high-risk group had a reduced survival time compared with those in the low-risk group. In addition, the AUCs of the classifier were 0.736 for 1-year overall survival and 0.682 for 3-year overall survival (**Figure 6C**).

These results indicate that the Mito-RGs-based classifier provides a useful prognostic tool with clinical value for appropriately categorizing patients with HCC.

### The Primary Bile Acid Biosynthesis Pathway Was Downregulated in High-Risk HCC Patients

**Figure 7:**
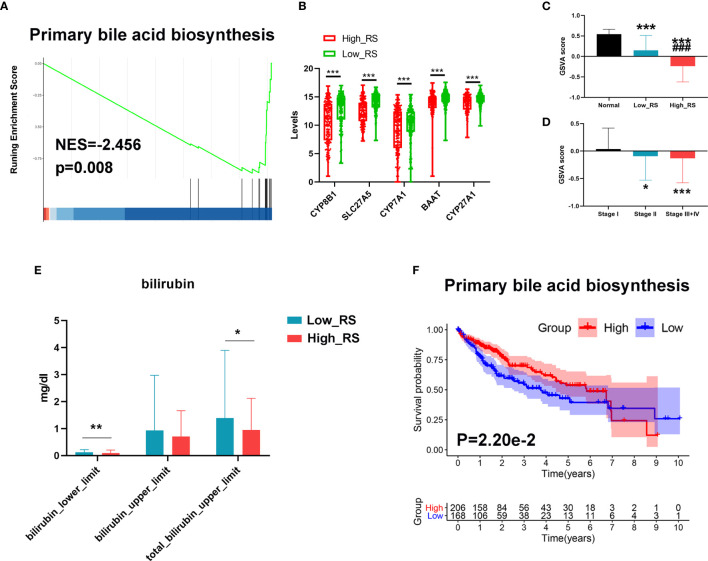
Changes in primary bile acid metabolic processes between high- and low-risk HCC. **(A)** The primary bile acid biosynthesis pathway was significantly enriched in low-risk HCC as revealed by GSEA analysis. **(B)** Top 5 downregulated genes of the primary bile acid biosynthesis pathway in high-risk HCC samples. **(C)** The primary bile acid biosynthesis pathway was significantly downregulated in high-risk HCC compared with low-risk HCC and normal liver tissues as revealed by GSVA analysis. ***P < 0.001 vs normal group; ^###^ *P* < 0.001 *vs* Low risk score. **(D)** The primary bile acid biosynthesis pathway was significantly downregulated in stage III+IV compared with stage II and stage I HCC as revealed by GSVA analysis. **(E)** The levels of bilirubin between low- and high-risk HCC. **(F)** Kaplan–Meier survival analysis of the prognostic value of altered metabolic pathways in HCC. GSVA score = 0 was set as the threshold value. *P < 0.05, ***P < 0.001 *vs* stage I.

Since bile acid is a liver-specific metabolic substance, we investigated a possible role for measurements of primary bile acid biosynthesis in prognosis of HCC. As shown in **Figures 7A, B**, primary bile acid biosynthesis was significantly higher in low-risk HCC, and key regulatory genes were downregulated in high-risk HCC. In addition, GSVA analysis showed that the primary bile acid biosynthesis pathway was significantly downregulated in high-risk HCC compared with low-risk HCC and normal liver tissue (**Figure 7C**). This pathway was also downregulated more in stage III+IV than in stage II or stage I HCC (**Figure 7D**). Moreover, the levels of bilirubin were also lower in high-risk compared with low-risk HCC (**Figure 7E**).

Finally, we conducted Kaplan-Meier analyses to further verify the value of these processes to the prognosis of HCC. As shown in **Figure 7F**, a high level of primary bile acid biosynthesis correlated with a favorable prognosis.

### Metabolic Subgrouping

**Figure 8:**
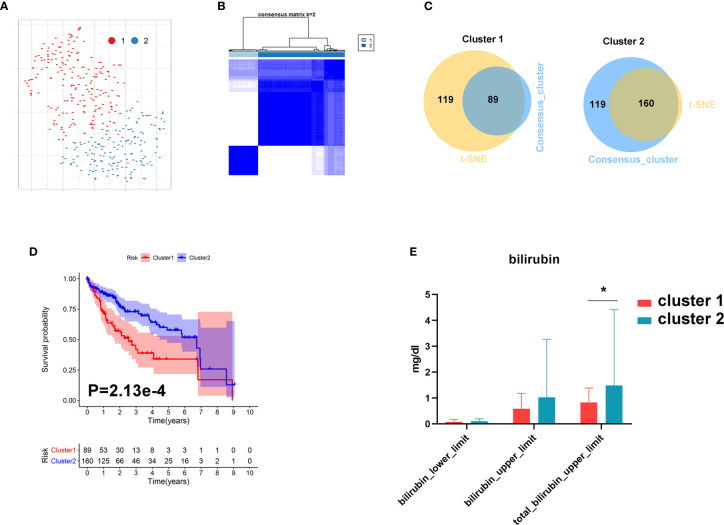
Metabolic subgroup of HCC based on primary bile acid biosynthesis. **(A)** Dot plot for two distinct clusters identified by t-SNE. **(B)** Heat map for two distinct clusters identified by consensus clustering solution. **(C)** Venn plot for identifying common samples in the two clusters. **(D)** Kaplan–Meier survival analysis for patients in two clusters. **(E)** The levels of bilirubin between the two clusters. *P < 0.05.

In order to further investigate the role of bile acid metabolism in HCC, we conducted cluster analysis based on gene expression of the primary bile acid biosynthesis pathway. As shown in **Figures 8A, B**, HCC tissues were clearly divided into two subgroups based on t-SNE and Consensus ClusterPlus software analysis. We further identified 89 samples in cluster 1 and 160 samples in cluster 2 (**Figure 8C**). We found that patients with a higher level of bilirubin in cluster 2 share a favorable prognosis (**Figures 8D, E**).

These results further demonstrate that metabolic processes governing bile acid biosynthesis affect the prognosis of HCC.

### Correlation Between Bile Acid Biosynthesis Pathways and Immune Cell Infiltration Into Tumors

The bar plots in **Supplementary Figure 2A** show the proportion of 22 immune cells in every sample. The five most common immune cells in HCC were resting CD4 memory T cells (24.6%), M2 macrophages (20.1%), M0 macrophages (8.3%), naive B cells (7.2%) and regulatory T cells (Tregs) (6.9%). The heat map of 22 immune cells is shown in **Supplementary Figure 2B**.

**Figure 9:**
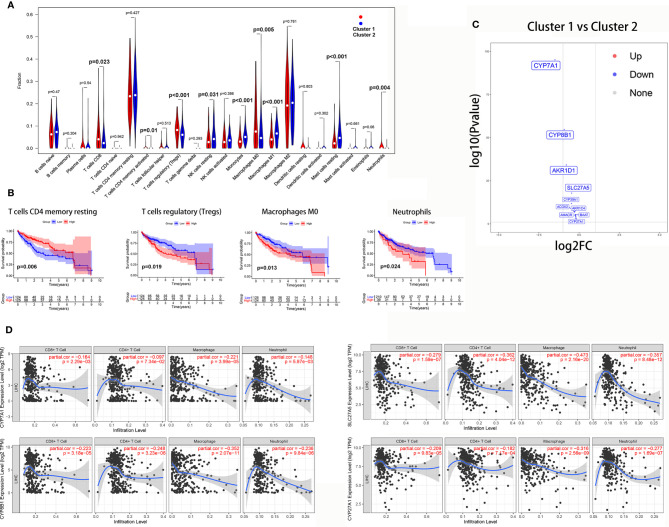
The correlation between the bile acid biosynthesis pathway and immune cell infiltration of tumors. **(A)** The comparison of the fractions of immune cells between the cluster 1 and cluster 2 HCC samples. **(B)** Kaplan-Meier survival analysis of overall survival between high and low levels of infiltrating immune cells. **(C)** Volcano plot of all genes in the primary bile acid biosynthesis between cluster 1 and cluster 2. **(D)** The correlation between gene expression and immune cell infiltration of tumors.

The Wilcoxon rank-sum test revealed that tumor-infiltrating CD8 T cells (P = 0.023), activated CD4 memory T cells (P = 0.01), Tregs (P<0.001), M0 macrophages (P = 0.005), and neutrophils (P = 0.004) were significantly higher in cluster 1. However, resting NK cells (P = 0.031), M1 macrophages (P<0.001), monocytes (P<0.001), and resting mast cells (P<0.001) were significantly higher in cluster 2 (**Figure 9A**).

Additionally, Kaplan-Meier analysis showed that patients with a high proportion of resting CD4 memory T cells exhibited greater overall survival (P = 0.006), while patients with a high proportion of Tregs (P = 0.019), M0 macrophages (P = 0.013), and neutrophils (P = 0.024) exhibited lower overall survival (**Figure 9B**).

To further investigate the relationship between the bile acid biosynthesis pathway and tumor infiltration by immune cells, the “edgeR” package of R software was used to detect the differentially expressed genes (DEGs) between clusters 1 and 2 from the HCC samples. As shown in **Figure 9C**, almost all genes in the primary bile acid biosynthesis pathway were downregulated in cluster 1. Furthermore, CYP7A1, CYP8B1, SLC27A5, and CYP27A1 there was a significant negative correlation with infiltration of CD8+ T cells, macrophages, and neutrophils (**Figure 9D**).

## Discussion

HCC is one of the leading causes of cancer-related deaths because it is highly malignant, recurrent, metastatic, drug-resistant, and usually diagnosed late in its progression (26). Thus, identification of effective biomarkers for HCC-specific prognosis is urgently needed to improve patient management. Recently, global changes in metabolic pathways were identified in HCC (27), providing new diagnostic and therapeutic opportunities (28). Taking into account the importance of changes in metabolic processes during HCC progression and the fundamental importance of mitochondria in human metabolism, it is essential to identify mitochondrial‐related biomarkers that can be used for prognosis of HCC patients. Such biomarkers may also help us to clarify underlying metabolic changes and identify potential therapeutic drugs to improve the prognosis of HCC patients.

In the present study, a 10 Mito-RGs-based prognostic classifier for HCC was constructed and validated for prognosis HCC patients for the first time. The classifier performed well in predicting the progression of HCC patients in the TCGA training and ICGC external validation cohorts, supporting the repeatability and utility of the classifier for prognosis of HCC overall survival. Furthermore, the prediction efficacy of the classifier was superior to those of histologic grade and tumor stage (TNM stage), which are two previously reported major risk factors for tumor progression (29, 30). Additionally, the prediction efficacy of the classifier was also superior to other predictive models of the progression of HCC.

All 10 Mito-RGs of the classifier, ACOT7, ADPRHL2, ATAD3A, BSG, FAM72A, PDK3, PDSS1, RAD51C, TOMM34, and TRMU, were risk-associated, and more highly expressed in the high-risk group. Among them, ACOT7, ADPRHL2, ATAD3A, BSG, FAM72A, PDSS1, RAD51C, and TOMM34 were overexpressed in HCC compared with normal liver tissues, indicating potential roles of these genes in the initiation and development of HCC.

ACOT7 is an isoform of the acyl-CoA thioesterase (ACOT) family, which is responsible for cleaving fatty acyl-CoAs to free fatty acids (31). High expression of ACOT7 is associated with unfavorable outcomes in acute myeloid leukemia patients (32). A previous study found that upregulation of ACOT7 was associated with metabolites that differ among chronic hepatitis B, liver cirrhosis, and HCC (33). These findings may provide clues to the mechanism by which ACOT7 affects the pathogenesis of HCC. ADPRHL2, also known as ADP-ribosylhydrolase 3 (ARH3), is the main hydrolase for catalyzing the hydrolysis of ADP-ribosylated serine. ARH3 is essential in negatively regulating parthanatos, a form of poly(ADP-ribose) polymerase 1 (PARP1)-mediated regulated cell death caused by excessive DNA damage (34). Induction of parthanatos is emerging as a new strategy to kill cancer cells (35). However, ARH3-overexpressing cells exhibit decreased PAR accumulation and PARP1-mediated cell death (35), indicating the potential carcinogenic role of ARH3. Until now, there have been few reports of ADPRHL2 in carcinogenesis, and the role and mechanism of ADPRHL2 in HCC need further investigation. ATPase family AAA domain-containing protein 3 (ATAD3A) is a mitochondrial membrane-bound ATPase involved in various cellular processes, including mitochondrial dynamics, lipogenesis, development, and cancer (36). Overexpression of ATAD3A has been observed in various types of cancer (37). The expression of ATAD3A is tightly correlated with the disease status, tumor grade, and lymphovascular infiltration in prostate cancer (38) and uterine cervical cancer (39). In addition, the high expression of ATAD3A is correlated with poor prognosis in lung cancer (40) and HCC (41). However, a recent study found that ATAD3A played important roles in reversing sorafenib resistance by mediating hypoxia-induced mitophagy signaling in HCC (42). To date, there are few reports on the carcinogenic effects of ATAD3A in HCC, and further experiments are needed to confirm its effect on HCC. Basigin (BSG), designated CD147, is a member of the immunoglobulin superfamily that is involved in various physiological functions, including carcinogenesis (43). Previous studies demonstrated that CD147 is highly expressed in various cancers, including those of the liver, kidney, colon, lung, breast, prostate, and esophagus (44). There is emerging evidence indicating that CD147 plays a central role in the progression and chemoresistance of many cancers by promoting proliferation, angiogenesis, migration, invasion, and anti-apoptosis (43, 45–47). Besides, multiple studies demonstrated that CD147 is overexpressed and positively correlated with HCC malignant potential and poor prognosis (48, 49). CD147 plays an important role in HCC invasion and metastasis, mainly *via* modulating fibroblasts and tumor cells themselves to disrupt the HCC microenvironment (50). FAM72A, also known as p17 or Ugene, is a novel neuronal protein that also exerts tumorigenic effects in multiple tissues (51). Previous studies demonstrated that FAM72A is highly expressed in multiple cancers, including liver cancer (52). In addition, FAM72A plays a central role in progression by accelerating the G1/S phase transition in the cell cycle and promoting the survival of cancer cells (53, 54). RAD51C is one of the paralogs of RAD51 and is essential for homologous recombination, a critical mechanism for DNA repair (55). Accurate DNA repair and replication are of importance to genomic stability and cancer prevention. RAD51C is thus involved in the development and progression of cancer. Previous studies found that high expression of RAD51C is associated with a poor prognosis and correlated with resistance to chemoradiotherapy in lung and breast cancers (56, 57). TOMM34 is a protein located in the outer membrane of mitochondria (TOMM), which plays a role in importing preprotein into the mitochondria (58). A previous study found that TOMM34 was overexpressed in colon cancer (59), ovarian cancer (60), and breast cancer (61) and served as a biomarker of the progression and poor prognosis of ovarian and breast cancer. However, to date, the role of ACOT7, ADPRHL2, ATAD3A, FAM72A, PDSS1, RAD51C, and TOMM34 in HCC almost unclear. Considering their strong relevance to the prognosis of HCC, the roles of these genes in HCC are worthy of further investigation.

Metabolic changes are a well-founded hallmark of cancers, including HCC (62). The wide range of metabolic alterations is strongly associated with the heterogeneity of HCC, providing challenges for clinical management of HCC patients (6). Bile acids are liver-specific metabolites derived from cholesterol. Primary bile acids are synthesized from cholesterol in the liver by classic and alternative pathways. The classic pathway accounts for about 90% of total bile acid production in the liver (cholic acid (CA) and chenodeoxycholic acid (CDCA)), mainly catalyzed by cholesterol 7α-hydroxylase (CYP7A1) (63). The alternative pathway is catalyzed by CYP27A1 and CYP7B1, which produces chenodeoxycholic acid (CDCA) (63).

Early in the 1970s, it was shown that plasma bile acid concentrations are elevated in HCC patients compared with healthy individuals (64), indicating that bile acid homeostasis was disturbed in HCC. However, the role of bile acid in carcinogenesis remains controversial. In recent years, evidence has accumulated in support of a crucial role for bile acids in gastrointestinal and hepatic carcinogenesis. Chronic and advanced-stage cholestasis patients may be at higher risk of developing HCC and bile duct cancer (65). The knockout of the Farnesoid X receptor (FXR), an endogenous ligand for bile acids, lead to elevation of bile acid concentration and resulted in development of liver tumors in mice (66, 67). Bile acids can directly disrupt the plasma membrane and activate the PKC-MAPK-NF-κB pathway, increasing TNF-α, IL-1β, and IL-6. These cytokines activate the JAK-STAT3 and PI3K-MDM2 pathways, which increase the survival of DNA-damaged cells and can lead to development of HCC. Besides, membrane injury by bile acids can also lead to an increase in reactive oxygen species (ROS) in hepatocytes by activating cytosolic phospholipase A2 (PLA2), which can directly activate NF-κB and also induce DNA damage in cells, which might lead to HCC (68). On the contrary, some studies have indicated that bile acids are tumor suppressors involved in the pathogenesis of HCC. A high concentration of bile acids induced cancer cell apoptosis by membrane disruption or the activation of caspase signaling (69). In addition, a high concentration of bile acids also inhibited cell proliferation and regeneration (70), which may slow the progression of HCC. Based on these findings, multiple synthetic bile acid derivatives have been designed and found useful for cancer therapy (71). In the present study, we found that high-risk HCC patients had a lower activity of the primary bile acid biosynthesis pathway than did low-risk patients. Furthermore, the primary bile acid biosynthesis pathway declined with an increase in tumor stage, indicating that activation of the primary bile acid biosynthesis pathway may serve as a tumor suppressor in HCC. However, further experiments are needed to validate the role and mechanism of primary bile acid biosynthesis pathway on HCC progression and prognosis.

A recent study found that bile acids can serve as messengers in the gut microbiome to control accumulation of hepatic NKT cells by upregulation of CXCL16 and anti-tumor immunity against both primary and metastatic liver tumors in the liver (72), suggesting the importance of bile acids in modulating the tumor immune microenvironment. In the present study, we first investigated the role of bile acids and tumor immune cell infiltration into tumor tissue and found that the numbers of tumor-infiltrating CD8 T cells, activated CD4 memory T cells, Tregs, M0 macrophages, and neutrophils were significantly higher in patients with low levels of bile acid biosynthesis. However, there were significantly higher numbers of resting NK cells, M1 macrophages, monocytes, and resting mast cells in patients with low levels of bile acid biosynthesis. High numbers of infiltrating Tregs, M0 macrophages, and neutrophils were correlated with a poor prognosis of HCC, indicating bile acid and its metabolites play important roles in HCC through regulating infiltration of tumors by Tregs, M0 macrophages, and neutrophils.

The immune-modulatory effects of bile acids have been widely researched in the gastrointestinal tract (68) and liver (73). Bile acid-activated receptors (BARs) including FXR and Takeda G-protein receptor 5 (TGR5) are highly expressed in innate immune cells such as dendritic cells (DCs), monocytes, macrophages, NK cells, and NKT cells (73). Activation of FXR and TGR5 in macrophages by bile acid leads to a polarization toward the anti-inflammatory M2 phenotype with IL-10 upregulation and downregulation of IL-6 and INF-γ. In the DCs, bile acid induced the downregulation of TNF-α and IL-12. In NKT cells, bile acid decreased the expression of IL-1β, TNF-α, and IFN-γ, resulting in a tolerogenic state of innate immunity in the liver and intestine (74). Furthermore, bile acids also promote inflammation by disrupting the plasma membrane, leading to activation of the PKC-MAPK-NF-κB pathway, and increasing production of TNF-α, IL-1β, and IL-6 (68). As for the role of bile acid on adaptive immunity, a recent study showed that the bile acid metabolites, 3-oxo lithocholic acid (LCA) inhibited Th17 differentiation by directly binding retinoid-related orphan receptor γt (RORγt), and isoalloLCA enhanced differentiation of Tregs through the production of mitochondrial reactive oxygen species (75).

The mechanism of bile acid-mediated immune cell infiltration of tumors remains unclear. In the present study, we found that the genes controlling primary bile acid biosynthesis, CYP7A1, CYP8B1, SLC27A5, and CYP27A1 negatively correlated with infiltration of CD8+ T cells, macrophages, and neutrophils. These results provide clues for further investigation. However, the mechanism by which these genes mediate infiltration of tumors by immune cells requires further exploration.

Inevitably, the present study has some limitations. Firstly, it was a retrospective study based on public online databases. Second, only two cohorts consisting of 606 samples were included. Therefore, large-scale, multi-center studies are needed to verify our results before the Mito-RGs-based classifier can be used in the clinic.

## Conclusion

In conclusion, we first identified and validated a classifier containing 10 Mito-RGs with independent prognostic significance for patients with HCC. Based on the classifier, we showed that the primary bile acid biosynthesis pathway was correlated with the prognosis of HCC, indicating that this pathway and related metabolites provide potential targets for anti-tumor treatments. Moreover, our research reveals cross-talk between bile acid and immune cell infiltration of tumors, which may provide novel insight into immunotherapy of HCC. Finally, our research may provide a novel method for HCC metabolic therapy based on modulation of mitochondrial function.

## Data Availability Statement

Publicly available datasets were analyzed in this study. This data can be found here: The Cancer Genome Atlas (https://portal.gdc.cancer.gov/) (TCGA-LIHC); the NCBI Gene Expression Omnibus (GSE76427 and GSE10143).

## Author Contributions

TZ and YN: design, analysis, and interpretation of data, and drafting of the manuscript. JG and KC: acquisition of data and statistical analysis. XC, HL, and JW: critical revision of the manuscript for important intellectual content, obtaining funding, and supervision. All authors contributed to the article and approved the submitted version.

## Funding

This work was supported by the National Natural Science Foundation of China (NO. 82070647, 81602419, and 81571075) and the National Key Research and Development Project (No. 2018YFC2001802).

## Conflict of Interest

The authors declare that the research was conducted in the absence of any commercial or financial relationships that could be construed as a potential conflict of interest.

## Supplementary Material

Supplementary Table 2 Gene list of the red module.

Supplementary Figure 1 Univariate Cox analysis for the Mito-RGs.

Supplementary Figure 2 The composition **(A)** and heat map **(B)** of immune cells estimated by CIBERSORT algorithm in HCC.

## Glossary

**Table:**

ACOT7	acyl-CoA thioesterase 7
ADPRHL2	ADP-ribosylhydrolase like 2
ATAD3A	ATPase family AAA domain containing 3A
AUC	area under the curve
BSG	basigin
CA	cholic acid
CDCA	chenodeoxycholic acid
CIBERSORT	cell type identification by estimating relative subsets of RNA transcripts
CXCL16	C-X-C motif chemokine ligand 16
CYP27A1	cytochrome P450 family 27 subfamily A member 1
CYP7A1	cytochrome P450 family 7 subfamily A member 1
CYP8B1	cytochrome P450 family 8 subfamily B member 1
DEGs	differentially expressed genes
FAM72A	family with sequence similarity 72 member A
FXR	farnesoid X receptor
GEO	gene expression omnibus
GO	gene ontology
GSEA	gene set enrichment analysis
GSVA	gene set variation analysis
HCC	hepatocellular carcinoma
HR	hazard ratio
ICGC	International Cancer Genome Consortium
JAK	Janus kinase 2
KEGG	Kyoto Encyclopedia of Genes and Genomes
LASSO	least absolute shrinkage and selection operation
LIHC	liver hepatocellular carcinoma
LIRI-JP	Liver Cancer-RIKEN-Japan
MAPK	mitogen-activated protein kinase
Mito-RGs	mitochondrial-related genes
NES	normalized enrichment score
OS	overall survival
PDK3	pyruvate dehydrogenase kinase 3
PDSS1	decaprenyl diphosphate synthase subunit 1
PI3K	phosphoinositide 3 kinase
PKC	protein kinase C
PLA2	cytosolic phospholipase A2
RAD51C	RAD51 paralog C
ROC	receiver operating characteristic
ROS	reactive oxygen species
SLC27A5	solute carrier family 27 member 5
STAT3	signal transducer and activator of transcription 3
TCGA	The Cancer Genome Atlas
TGR5	takeda G-protein receptor 5
TIMER	tumor immune estimation resource
TOM	topological overlap matrix
TOMM34	translocase of outer mitochondrial membrane 34
TRMU	tRNA 5-methylaminomethyl-2-thiouridylate methyltransferase
t-SNE	t-distributed Stochastic Neighbor Embedding
WGCNA	weighted gene coexpression network analysis
