# Corrigendum: Identification of Mitochondrial-Related Prognostic Biomarkers Associated With Primary Bile Acid Biosynthesis and Tumor Microenvironment of Hepatocellular Carcinoma

**DOI:** 10.3389/fonc.2021.843623

**Published:** 2022-01-17

**Authors:** Tao Zhang, Yingli Nie, Jian Gu, Kailin Cai, Xiangdong Chen, Huili Li, Jiliang Wang

**Affiliations:** ^1^ Department of Anesthesiology, Union Hospital, Tongji Medical College, Huazhong University of Science and Technology, Wuhan, China; ^2^ Department of Dermatology, Wuhan Children’s Hospital (Wuhan Maternal and Child Healthcare Hospital), Tongji Medical College, Huazhong University of Science and Technology, Wuhan, China; ^3^ Department of Gastrointestinal Surgery, Union Hospital, Tongji Medical College, Huazhong University of Science and Technology, Wuhan, China

**Keywords:** hepatocellular carcinoma, mitochondria, prognosis, bile acid, tumor microenvironment

There is an error in the Funding statement. The correct numbers for the National Natural Science Foundation of China are NO. 82070647, 81602419, and 81571075.

The authors apologize for this error and state that this does not change the scientific conclusions of the article in any way. The original article has been updated.

## Publisher’s Note

All claims expressed in this article are solely those of the authors and do not necessarily represent those of their affiliated organizations, or those of the publisher, the editors and the reviewers. Any product that may be evaluated in this article, or claim that may be made by its manufacturer, is not guaranteed or endorsed by the publisher.

