# Supplementary material for: Identification of Mitochondrial-Related Prognostic Biomarkers Associated With Primary Bile Acid Biosynthesis and Tumor Microenvironment of Hepatocellular Carcinoma
Source: Front Oncol. 2021 Apr 1;11:587479. doi: 10.3389/fonc.2021.587479 (PMC8047479; doi:10.3389/fonc.2021.587479)
Supplement: Supplementary Table 2 — Gene list of the red module. [file Table_2.docx]

| **Module** | **Genes** |
| --- | --- |
| Red  (n=66) | ABCC8, ABHD11, ACOT7, ACSBG2, ADPRHL2, AIFM3, ANKZF1, ATAD3A, ATAD3B, ATP2A1, BAK1, BBC3, BMF, BSG, CAPN10, CCNB1, CDK1, COA1, COX19, DLGAP5, DTYMK, E2F1, FADS1, FAM72A, FANCG, FEN1, FLVCR1, FUNDC1, FUNDC2, GARS, GNRH1, GPS2, HJURP, HKDC1, KCNJ11, LIG1, LIPT2, MRM2, MRPL18, MRPL53, MTFR2, NDUFA4L2, NIPSNAP2, NUDT1, OGG1, PDK3, PDSS1, PIF1, PRELID2, RAD51, RAD51C, SLC25A45, TOMM34, TOMM5, TP73, TRIM31, TRMU, TYMS, VAT1, XRCC3, YJEFN3, YKT6, AC006538.1 |
