# Supplementary figures and images for: Identification of Mitochondrial-Related Prognostic Biomarkers Associated With Primary Bile Acid Biosynthesis and Tumor Microenvironment of Hepatocellular Carcinoma
Source: Front Oncol. 2021 Apr 1;11:587479. doi: 10.3389/fonc.2021.587479 (PMC8047479; doi:10.3389/fonc.2021.587479)

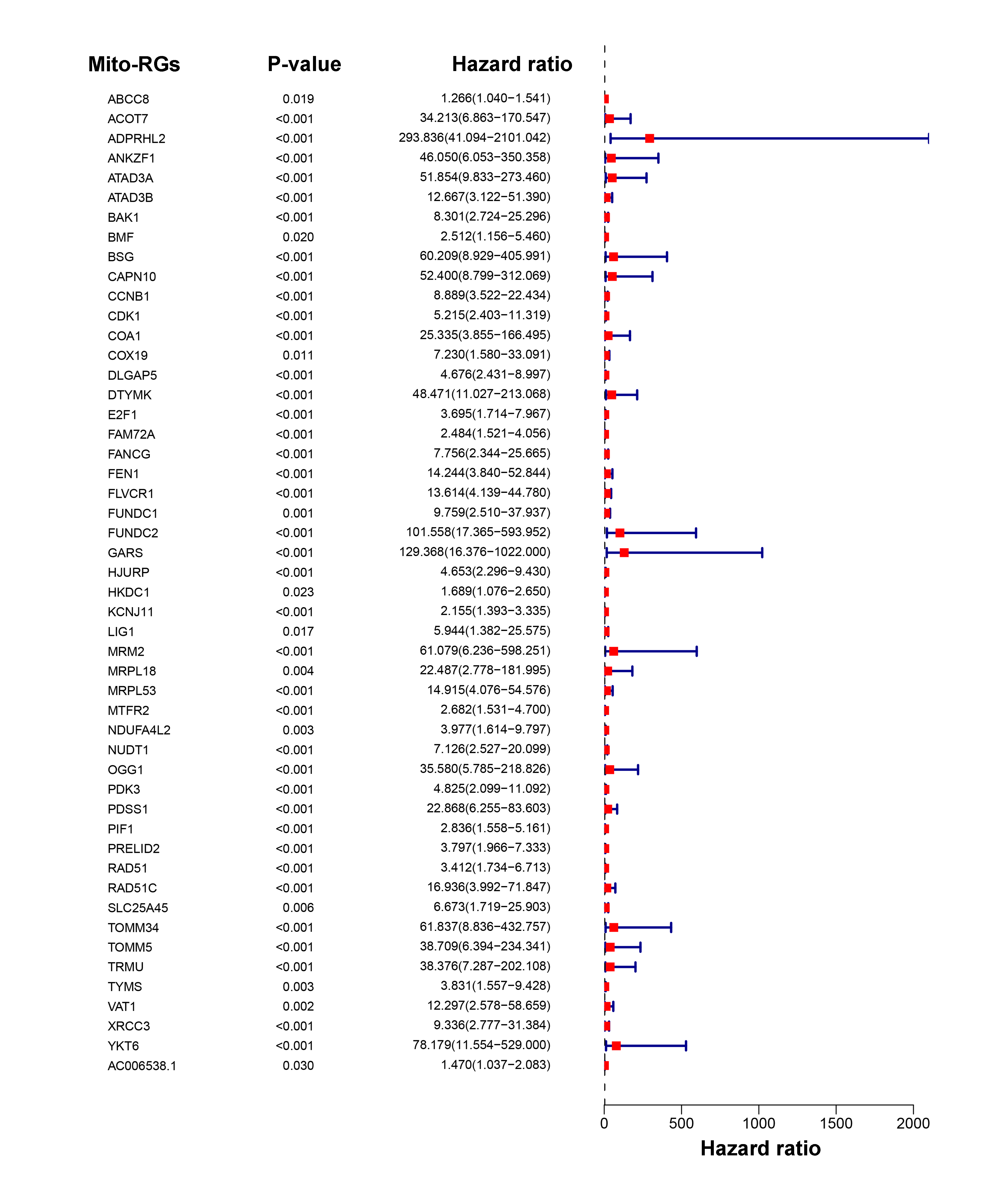

Supplement: Supplementary Figure 1 — Univariate Cox analysis for the Mito-RGs. [file Image_1.tif]

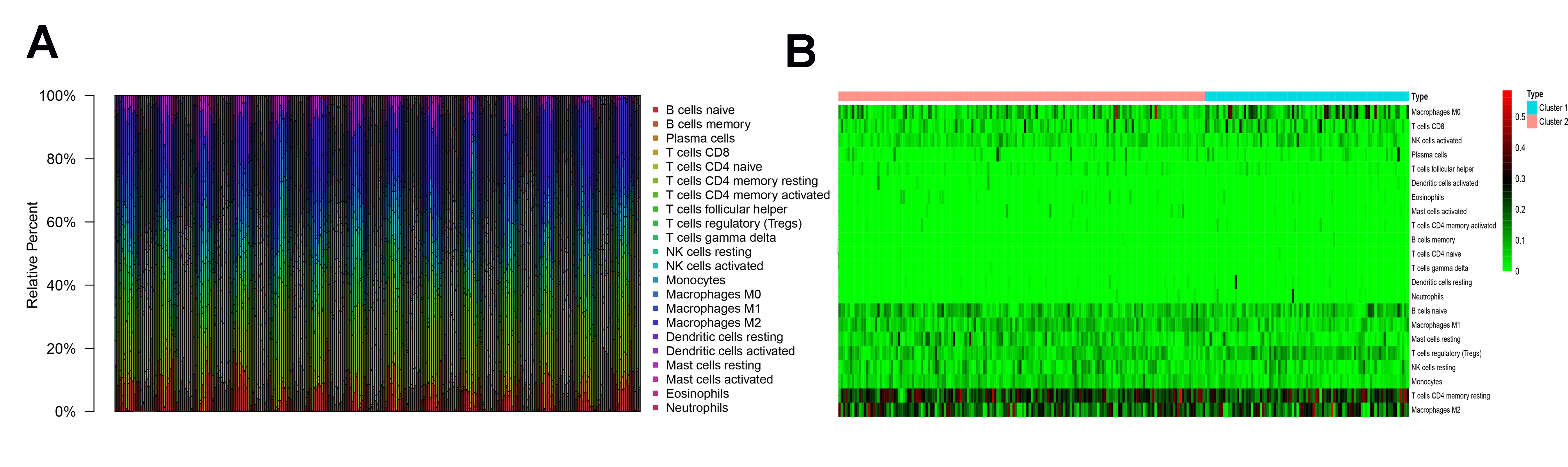

Supplement: Supplementary Figure 2 — The composition (A) and heat map (B) of immune cells estimated by CIBERSORT algorithm in HCC. [file Image_2.tif]
